# Assessing shortfalls and complementary conservation areas for national plant biodiversity in South Korea

**DOI:** 10.1371/journal.pone.0190754

**Published:** 2018-02-23

**Authors:** Hyeyeong Choe, James H. Thorne, Patrick R. Huber, Dongkun Lee, James F. Quinn

**Affiliations:** 1 Department of Environmental Science and Policy, University of California, Davis, California, United States of America; 2 Department of Landscape Architecture & Rural System Engineering, College of Agriculture Life Sciences, Seoul National University, Seoul, South Korea; Leiden University, NETHERLANDS

## Abstract

Protected areas (PAs) are often considered the most important biodiversity conservation areas in national plans, but PAs often do not represent national-scale biodiversity. We evaluate the current conservation status of plant biodiversity within current existing PAs, and identify potential additional PAs for South Korea. We modeled species ranges for 2,297 plant species using Multivariate Adaptive Regression Splines and compared the level of mean range representation in South Korea’s existing PAs, which comprise 5.7% of the country’s mainland area, with an equal-area alternative PA strategy selected with the reserve algorithm Marxan. We also used Marxan to model two additional conservation scenarios that add lands to approach the Aichi Biodiversity Target objectives (17% of the country). Existing PAs in South Korea contain an average of 6.3% of each plant species’ range, compared to 5.9% in the modeled equal-area alternative. However, existing PAs primarily represent a high percentage of the ranges for high-elevation and small range size species. The additional PAs scenario that adds lands to the existing PAs covers 14,587.55 km^2^, and would improve overall plant range representation to a mean of 16.8% of every species’ range. The other additional PAs scenario, which selects new PAs from all lands and covers 13,197.35 km^2^, would improve overall plant range representation to a mean of 13.5%. Even though the additional PAs that includes existing PAs represents higher percentages of species’ ranges, it is missing many biodiversity hotspots in non-mountainous areas and the additional PAs without locking in the existing PAs represent almost all species’ ranges evenly, including low-elevation ones with larger ranges. Some priority conservation areas we identified are expansions of, or near, existing PAs, especially in northeastern and southern South Korea. However, lowland coastal areas and areas surrounding the capital city, Seoul, are also critical for biodiversity conservation in South Korea.

## Introduction

Biological diversity contributes to food security, human health, the provision of clean air and water, and enhancement of local livelihoods and economic development [1]. However, species extinction is progressing rapidly, and biodiversity continues to be lost [2–5]. Efforts to halt this crisis are ongoing, but limited funds influence the conservation strategies and planning methods that can be used [6]. Designation of additional conservation areas could help maintain overall biodiversity [7], but could perhaps be more effective if conducted at broader scales, such as at the national or global scales [8,9], which require assessment of entire landscapes to identify proposed new conservation areas [10].

Protected natural areas (PAs) such as national parks are often the most important component of national conservation policies. However, PAs are usually not representative of national biodiversity [11]. Gap analysis has been used to measure effectiveness of existing PAs in representing all elements of regional biodiversity, with the goal of identifying gaps in protection and optimizing new PA acquisitions [12]. Such analyses often use species occurrence records to assess representation [13], however, many such data collections have problems including incompleteness and geographical biases [14]. Thus, some efforts to identify additional conservation areas have had to rely on limited species data or surrogate information. Examples include Bolliger *et al*. [15], who identified highly probable areas of species occurrence based on species habitat suitability modeling for seven animal species and designated the upper 10% of species’ ranges suitability areas as core habitats; and Hall *et al*. [16], who estimated the potential biodiversity values of patches based on land cover type, landscape position, patch size, and distance to mature forest patches.

When more species data are available, reserve decision support programs such as Marxan or Zonation [17] can be used to determine priority of conservation areas [18,19]. For example, Scherer *et al*. [20] predicted alpha and beta diversity patterns using 1,893 plant species in Kenya and used Zonation to search for potential expansions of Kenya’s PAs. When species location records are available, Species Distribution Models (SDM; [21]) can be employed as inputs for Marxan. Zhang *et al*. [22] used 2,319 woody species ranges modeled in the Maxent SDM to estimate the species richness and then used Marxan to locate potential conservation areas. Similarly, Naoe *et al*. [23] used Maxent for the range modeling of 97 bird species, and then used the Marxan to identify candidate PAs. These two studies used species modeled with five or more records.

We used data from a large national survey to assess terrestrial plant species representation status of current existing PAs and to identify potential additional PAs. The national survey data contain occurrence records for 56% of all South Korean plant species. We modeled plant species distributions using climatic and topographic predictor variables for 2,297 plant species with the MARS (Multivariate Adaptive Regression Splines) multi-response SDM algorithm [24,25], and assessed biodiversity representation in the existing PAs as the mean proportion of plant species’ predicted ranges that were included in existing conservation areas. We assessed the current conservation status of four plant groups: 1) all species; 2) endangered species; 3) endemic species; and 4) biological resources species within the existing PAs.

We then used Marxan to create three alternative conservation scenarios. The first scenario asks whether an alternative spatial arrangement of PAs using the same area as existing PAs could increase conservation performance. The second and third scenarios select a higher percentage of the country for PAs, to represent conservation levels that are closer to the Aichi Biodiversity Targets ([1], target 11). Marxan may be used to identify the most essential potential PAs (“irreplaceable”) to include a reserve network by varying starting conditions and counting the proportion of times that each planning unit is part of the calculated network [26]. We suggest achievable priority conservation areas for national plant biodiversity conservation in South Korea by identifying the locations commonly selected from the final Marxan results.

## Materials and methods

**Fig 1:**
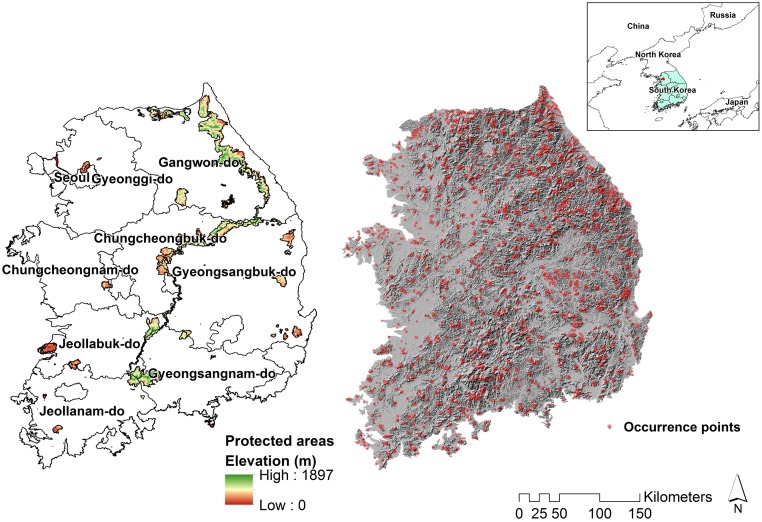
Distribution of the protected areas with province names (left) and locations of surveyed occurrence points of South Korea (right).

South Korea’s climate is temperate and affected by the East Asian monsoon, with four seasons (Fig 1). Mountains cover around 70% of the country, which has a population of 48.0 million [27]. The primary mountain range stretches along the east coast of South Korea, and mountains interface with southern and western coastal plains. This study was confined to the mainland of South Korea (95,219 km^2^) and excludes 4,814 km^2^ of islands. Various types of PAs cover 5,463.20 km^2^ (5.74% of the study area, as defined by GIS files of South Korean PAs from the Protected Planet website (http://www.protectedplanet.net/), the online interface for the World Database on Protected Areas).

**Fig 2:**
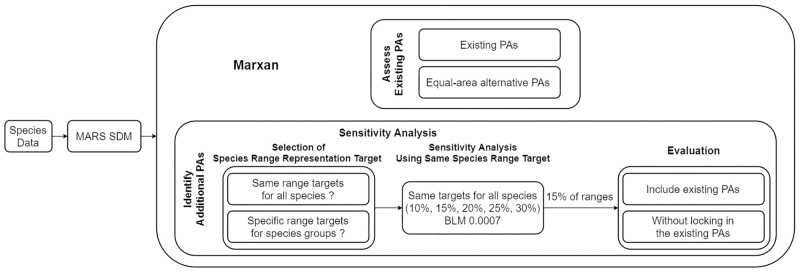
Study flow diagram. After we ran the MARS SDM to model 2,297 plant species ranges, we used Marxan for running alternative conservation scenarios: 1) Marxan generated PAs with a total area equal to existing PAs; 2) potential additional PAs to increase the representation of plant species in two ways. We considered two extent targets that increase overall PAs: a doubling of the existing PAs’ area and an increase to 17% of the mainland area. We selected additional PAs to closely reach to these extent targets from the top scored areas of the summed solution of 100 Marxan runs for each case.

There were four main steps: 1) Gather vascular plant species occurrence records and climate and environmental data for South Korea; 2) Run the MARS SDM to model species ranges; 3) Compare the existing PAs for plant biodiversity representation with the equal-area modeled alternative PAs; and 4) Identify additional conservation priority areas for national plant biodiversity conservation by using Marxan under alternative species range conservation target scenarios (Fig 2). We considered two extent targets that increase overall PAs: a doubling of the existing PAs’ area and an increase to 17% of the mainland area, the Aichi Biodiversity Targets ([1], target 11), of which South Korea is a signatory. However, Marxan does not select specific target area extents. It selects area based on the proportion of species range representation targeted for each species. We therefore selected additional lands from the top scored areas of the summed solution of 100 Marxan runs until the area closely matched the extent targets for each case in our study. We used the Marxan ‘‘irreplaceability” scores for planning units, and selected the target extents in descending order from the highest ranked location [28].

### Species and environmental data

We used 150,959 occurrence records for terrestrial plants, comprising 2,297 species from the Third National Ecosystem Survey data (S1 Table; National Ecosystem Survey data are available at http://ecobank.nie.re.kr). These observations were recorded over seven years by a large crew of biologists at 7,425 systematic grids across the country. The survey divides the country into a grid of 7,425 cells of 17.3 km^2^. A representative mountain area in each grid was selected to survey for plant species. A linear path over the mountain was walked to collect plant data, and the route was selected to find as many plant species as possible. Each observation point represents the location record for a single point observation [29]. An additional 1,833 species from the national plant species list do not appear in the inventory at all [30], meaning the survey documents 56% of known plant species. The number of occurrence records for each documented species varies from two to 762. The average number of occurrence points is 66. 891 species have fewer than 10 occurrence points and 560 species have fewer than five occurrence points. Some of these species with few points are not rare species.

The South Korean Wildlife Protection and Management Act [31] designates 77 vascular plant species as endangered, of which 14 are identified by 112 points in the national survey. In addition, 127 endemic species which occur only within South Korea are identified by 4,590 survey points. South Korea also designates 676 plant species which are highly worthy of protecting for conservation of biological diversity as ‘biological resources’ [32]. They are subject to the approval of outbound transfer, in consultation with the head of the relevant administrative agency. In our data, 364 of these plant species are recorded by 16,130 records.

We used climatic and topographic predictor variables in developing species range models. Climatic variables are important factors that influence plant species’ distribution [33]. For climate data, we obtained Bioclimatic variables (annual mean temperature, temperature seasonality, mean temperature of the warmest and coldest quarter of the year, annual precipitation, precipitation of the wettest and driest quarter of the year) for current time representing 1950–2000 from the WorldClim website ([34]; http://www.worldclim.org/). The Bioclimatic variables derived from monthly temperature and rainfall values were developed as potentially ecologically meaningful predictors and consist of 19 variables [33,34]. Topography can influence local climate conditions for plants by varying sunlight, soil moisture, and nutrients [35]. Topographic data (elevation and slope) were obtained from the Korean Water Management Information System (WAMIS), and we calculated aspect and northness (the sine of aspect) [36].

### MARS multi-response SDM

We used the MARS [25] multi-response SDM to model the plant species distributions. A multi-response model is appropriate because our data include many species with few records [37]. Multi-response SDMs combine all species data and use information on the presence of other species to supplement information for the modeled species. The MARS algorithm considers interactions between variables locally, and selects predictors using the signal from many species automatically. Knots, points where the coefficients of predictor variables change within the range of each predictor variable, are used to select relevant ranges for each selected predictor, and a linear response is fitted for each section [38–40]. Multi-response MARS requires a site-by-species matrix for fitting models, so locations of other species modeled simultaneously are considered as ‘inventory pseudo-absences’ [25].

In our MARS SDM test runs, MARS models concurrently modeling more than 300 species identified only one predictor variable or did not converge. To make the models computationally tractable, we divided the 2,297 species into 15 groups, and ran the MARS models for each group separately. Species for each group were randomly selected, and groups were composed of 153 or 155 species [41]. The number of observations within groups varied from 8,503 to 12,862. Model performance for each species was assessed using the area under the receiver operating characteristic curve (AUC), which ranges from 0 to 1, because AUC is an useful indicator of model accuracy applicable to SDMs for low-prevalence presence-only data [42], even though there are some criticisms [43]. A score of 0.5 implies the predictive discrimination of a model is no better than random, while a score of 1 indicates perfect discrimination [38,42]. AUC was calculated using model fit because many species have less than 10 points and we do not have an independent observation data set to provide validation.

We used the ‘raster’ [44] package in R (version 3.1.1) to extract environmental values from each occurrence point and to predict occurrence probabilities of each species in the study area, and the ‘mda’ [45] package to run the MARS models. We used Elith and Leathwick’s codes [25] to constrain predictions to fall between 0 and 1.

A threshold (or cut-off) is required to transform occurrence probabilities from the model results into binary presence or absence that can represent potential species’ ranges. Since many species have a few presence points and models were fit using ‘inventory pseudo-absences’, we used a data-driven approach to select species range thresholds [46]. We found that setting the threshold at one standard deviation of the occurrence probabilities among each species’ set of occurrence points performed better than the max SSS threshold (maximizing the sum of sensitivity and specificity) in a previous study with incomplete data sets [24]. We adopted this value as the species range threshold for each species to generate binary range maps [47].

### Marxan parameters for alternative conservation scenarios

We used Marxan 2.43 [48,49] for running alternative conservation scenarios: 1) an equal-area alternative PAs as existing PAs; and 2) potential additional conservation areas to increase the representation of plant species. We used a 4 km^2^ grid to represent planning units in all scenario simulations. The “Planning Unit versus Species” file includes the area of each species’ range within each planning unit. We chose the simulated annealing algorithm, which is an optimization method based on the iterative improvement with randomly chosen planning units [48,50], and each of the Marxan scenarios consisted of 100 repeat runs resulting in a summed solution score of 0–100.

#### Equal-area alternative using the existing PAs extent

To compare the protection provided to the modeled plant species by the existing PA network in South Korea with what might be achieved by a more strategically designed PA network, we conducted four scenario runs using Marxan. We investigated sensitivity of the outcomes to Marxan parameters by using two levels of species range’ representation and two boundary length modifiers (BLM). Increasing BLM values produce more spatially contiguous recommended PA networks, and are used to reflect the general preference for selecting networks of larger land units to enhance long-term population viability, avoid edge effects, and lower management costs. We chose two levels of species range representation: 1) 10% of each species’ range area and 2) 50% of the range area for each endangered species because they require intensive management, 30% of the range for each endemic and biological resources species because they are important species for biodiversity conservation, and 10% of the range area of other species (S2 Table). In our Marxan test runs, we found that using a 0.0007 BLM with 4 km^2^ grid planning units produced a perimeter/area ratio similar to that of the existing PAs, so we used two BLM options, 0 and 0.0007. The existing PAs were not locked in (status of all planning units = 0) in these four Marxan runs. We selected the same area as the existing PAs (5,463.2 km^2^) from the highest scores of the summed solution of 100 Marxan runs from each of the four tests to compare the results with the existing PAs.

#### Additional PAs

To identify additional potential PAs, we considered two extent targets: twice the existing PAs extent (10,926.08 km^2^, 11.47% of the mainland area), and 17% of the total mainland area (16,187.23km^2^). However, Marxan could not select specific target area extents. Therefore, we selected the nearest area extents to our conservation target extents from the top scored area of the summed solution of 100 Marxan runs. The existing PAs were locked in (status of existing PAs = 2, otherwise 0) for these runs.

We used two sensitivity analyses for this scenario: First, for the levels of species range representation, we compared uniform targets for all species (10% and 15% of the range area of each species) against varying range representation targets for specific groups: (1) 50% of the range area of endangered species, 30% of endemic species and biological resources, and 10% of remaining species; and (2) 55% of the range area of endangered species, 35% of endemic species and biological resources, and 15% of remaining species. We also applied two BLM options, 0 and 0.0007. We found that the PAs from the Marxan runs using uniform species range conservation targets for all species and the 0.0007 BLM produced higher representation of species’ ranges in the resulting simulated PAs (S3 Table). We therefore used uniform species range conservation targets for all species in increments of 5% between 10%-30% range representation (and with BLM = 0.0007) in the Marxan runs to identify extending PAs for South Korea (S4 Table).

### Protected areas additionality

In the final steps, we evaluated the Marxan-generated PAs by calculating the average percentages of species’ range captured for four species groups: 1) all species; 2) species recognized nationally as endangered; 3) endemic species; and 4) biological resource species [32]. In addition, we ran Marxan without locking in the existing PAs (status of all planning units = 0) using the same options as the Marxan run that produced the highest representation of species’ ranges in all species and for the specific species groups. We conducted this scenario to test for spatial bias, in case the Marxan runs including existing PAs tend to select new PAs near existing PAs and to potentially underrepresent important species rich areas that are far from existing PAs. We compared the elevation distributions of alternative PAs, the average percentages of species’ ranges captured as a function of range size, and the average percentages of species’ range captured as a function of elevation. We applied the Wilcoxon rank-sum test to compare the difference between two PA alternatives for each comparison class. We present the Marxan-selected PAs and the commonly selected areas to suggest priority conservation areas for national plant conservation.

## Results

### MARS SDM model

In the 15 MARS models runs, a small set of predictors plays a dominant role in explaining the probability of occurrence for the plant species in South Korea. Environmental predictor variables selected in the final models were annual mean temperature (13 out of 15 models), elevation (6/15), mean temperature of warmest quarter (3/15), annual precipitation (2/15), temperature seasonality (2/15), and mean temperature of coldest quarter (1/15) (S5 Table). The Area Under the receiver operating Characteristic curve (AUC) using ‘inventory pseudo-absences’ ranges from 0.16 to 0.99. The average AUC score was 0.70 (SD = 0.13) (S1 Table).

When we combined all species’ range models to identify the general distribution patterns of each species group, the highest species richness areas are in central western and central parts of South Korea, and in the eastern part of Jeollabuk province (max. 1,593 species) (S1A Fig). Endangered species richness is highest around central South Korea (northern & eastern part of Gyeongsangbuk province), and in coastal areas of Chungcheongnam and Jeollabuk provinces (10 species) (S1B Fig). Endemic and biological resources species richness are highest in the northern part of Gyeongsangbuk province and the eastern part of Jeollabuk province (80 and 227 species, respectively) (S1C and S1D Fig, province names in Fig 1). We provide each raster layer of S1 Fig in S1, S2, S3 and S4 Appendices.

### Plant biodiversity conservation status in the existing PAs

We selected the equal-area alternative PAs targetting 10% of the total range of each species, which produced the highest species range representation among the four scenario runs (S2 Table). The average percentage of species’ ranges in existing PAs was: 6.3% for all species, 14.4% for endangered species, 9.9% for endemic species, and 8.7% for biological resources species, which is higher than the proportion of ranges captured in the equal-area alternative PAs (Table 1). The average percentage of species’ ranges in the equal-area alternative PAs was: 5.9% for all species, 5.3% for endangered species, 5.1% for endemic species, and 5.2% for biological resources species (Table 1). The overlap of the equal-area alternatives with the existing PAs is quite low, at 5.9%.

**Table 1:** Average percentage of species’ ranges captured in the existing protected areas and in alternative PAs scenarios from Marxan results.

Scenario	Species range conservation target	Boundary length modifier	% of PAs to total land area	Mean % of all species’ ranges inside	Mean % of endangered species’ ranges inside	Mean % of endemic species’ ranges inside	Mean % of biological resources species’ ranges inside
Existing PAs	-	-	5.7	6.3	14.4	9.9	8.7
Equal-area alternative	10% of all species	0	5.1	5.9	5.3	5.1	5.2
Additional PAs including existing PAs	15% of all species	0.0007	15.3	16.8	30.2	22.4	20.8
PAs without locking in the existing PAs	15% of all species	0.0007	13.9	13.5	15.3	14.4	14.1

### Potential additional PAs

**Fig 3:**
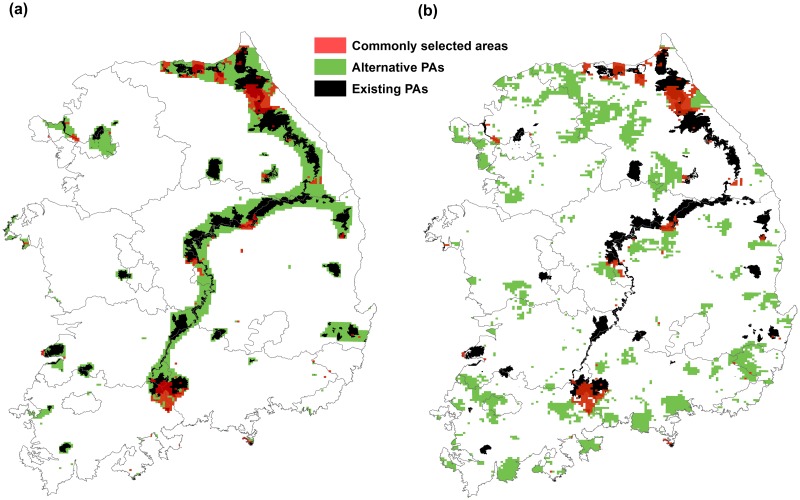
Alternative PAs from Marxan runs: (a) Additional PAs including the existing PAs (15.3% of total land area), (b) PAs without locking in the existing PAs (13.9% of total land area). Both Marxan runs targeted representing 15% of all species’ ranges, and the same BLM value applied to both Marxan runs.

For the additional PAs Marxan selected using uniform targets to all species, ranging from 10% to 30%, additional PAs are usually expansions surrounding existing PAs, particularly in the northeastern part of South Korea for all Marxan runs (Fig 3a). Compared to the representation of four specific species groups’ ranges, we found that the Marxan runs targetting 15% of all species’ ranges produced the PAs representing the highest percentages of species’ ranges for the four species group targets (S4 Table and S2 Fig). The average percentage of species’ ranges of PAs selected for 15.3% of total mainland area was: 16.8% for all species, 30.2% for endangered species, 22.4% for endemic species, and 20.8% for biological resources species (Table 1).

We ran Marxan targetting 15% of all species’ ranges without locking in the existing PAs to compare and evaluate the additional PAs (Fig 3b). The selected PAs for the amount of 13.9% of total mainland area were distributed largely across the northeastern part of South Korea and also in lowland coastal regions, particularly the south coast, where few existing PAs are located. The average percentage of species’ ranges of PAs selected for the amount of 13.9% of total mainland area was: 13.5% for all species, 15.3% for endangered species, 14.4% for endemic species, and 14.1% for biological resources species (Table 1).

**Fig 4:**
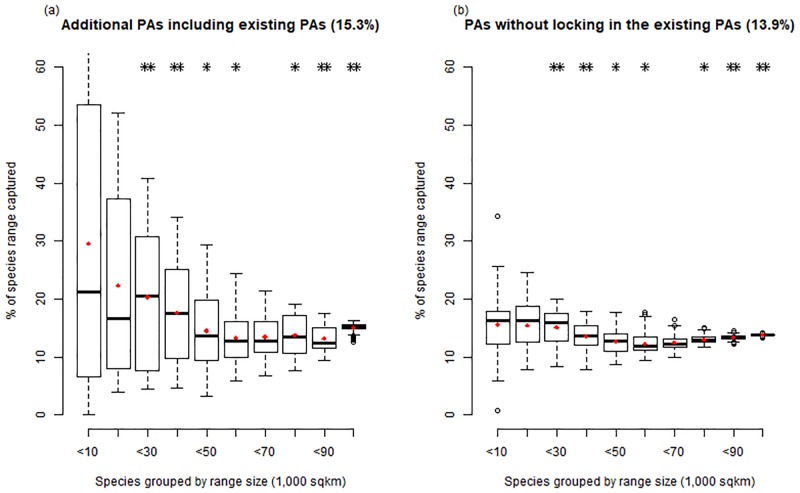
Comparisons between (a) additional PAs including existing PAs and (b) PAs without locking in the existing PAs as a function of species’ range size. The percentage of species’ ranges captured in each PAs as a function of species’ range size. Species were grouped by range size into 10 classes. We applied the Wilcoxon rank-sum test for each range size class to compare the difference between the two (* P ≤ 0.05; ** P ≤ 0.01). Representation in additional PAs including existing PAs is higher for species that range sizes are less than 10,000 km^2^. Red point in each bar is the average percentage within group.

**Fig 5:**
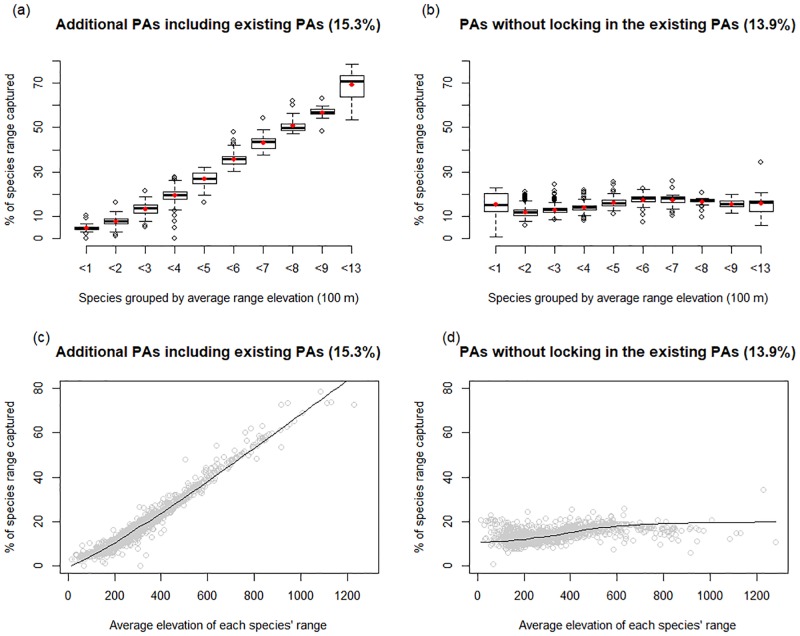
Comparisons between (a) additional PAs including existing PAs and (b) PAs without locking in the existing PAs as a function of average range elevation. In Figs 5a and 5b, species were grouped as a function of the average elevation of species’ range. We applied the Wilcoxon rank-sum test for each elevation class to compare the difference between the two (P ≤ 0.01 for all 10 elevation classes). Representation in additional PAs including existing PAs is significantly higher for species whose average elevation is higher than 200 m. The red point in each bar is the average percentage within group. In Figs 5c and 5d, species’ range captured in PAs were compared by species’ mean elevation ranges.

The existing PAs tend to be composed of high mountainous areas, with an average elevation of 558 m (SD = 320). The additional PAs selected by Marxan, plus the existing PAs, totaling 15.3% of total mainland area, produced mean elevations of 489 m (SD = 309), as compared to the mean elevations of 260 m (SD = 242) from the Marxan selected PAs without locking in the existing PAs (13.9% of total mainland area). Given the analysis that calculating the average percentage of species’ ranges by range size and by range elevation, the Marxan selected PAs without locking in the existing PAs represent almost all species’ ranges evenly, neither small-range species nor high-elevation species exclusively (Figs 4 and 5). Lowland areas are under-represented in the existing PAs of South Korea.

### Protected areas additionality

There are tradeoffs between the additional PAs scenarios that included or did not lock in the existing PAs. The Marxan run that targeted 15% of all species’ ranges and included the existing PAs captured 30.2% of the species ranges for endangered species and a higher percentage, 16.8%, for all species. By contrast, the same target run that started without existing PAs produced a lower percentage of species range representation, 13.5% for all species. However, this run captured a more even distribution of species ranges in lower elevations.

Areas that were commonly selected from the alternative PAs scenarios represent high priority conservation areas (Fig 3). Many commonly selected areas are near existing PAs, especially around northeastern and southern South Korea. Commonly selected areas are also distributed along the west and south coast areas.

The extent of commonly selected areas is 1,821 km^2^ including the parts of the existing PAs. The summed extent of these areas with the existing PAs is 6,192.47 km^2^, which is 6.50% of the South Korean mainland area.

## Discussion

Existing PAs outperformed the equal-area alternative PAs in the representation of plant species ranges. This is because existing PAs are mainly distributed in high elevation areas. The many high elevation plant species are well represented in the existing PA system and tend to give the existing PAs a higher range representation, even while many low elevation species ranges are not included.

For the scenarios that added area, the scenario that added to existing PAs captured a higher proportion of species ranges, but mainly distributed in high-altitude areas, while the scenario that added area starting with no PAs produced a more distributed allocation of hypothetical conservation areas including low elevation and coastal mountains. In both of these paired comparisons, the scenarios starting with no PAs identify types of landscapes that are currently under-represented in South Korea’s PAs.

The lower percentage of species ranges captured in the alternative PAs (Fig 3b) is likely due to the existing PAs that represent high proportions of the ranges of many plant species that occur at high elevations, while the alternative PAs that represent almost all species’ ranges evenly, not high-elevation species exclusively (Figs 4 and 5). The alternative PAs (Fig 3b) captured more species, but less of their range because it included many species from lower elevations that have larger ranges.

Marxan runs that were additive to existing PAs also identified low elevation areas as important complementary elements to the existing reserves, and we conclude that the existing PAs do not represent national-scale plant biodiversity for lowland species and that additional PAs in lowland areas in the southern and western parts of South Korea, including around the capital city, Seoul are needed (Fig 3).

Marxan is mainly intended to optimize conservation target levels for selected individual species and biodiversity features [51]. In this case Marxan was used to assess range representation of 2,297 plant species, which allowed us to identify additional priority conservation areas that would improve overall biodiversity representation (Table 1 and S2 Fig).

It may be difficult for small-sized countries to achieve the Aichi targets and designate 17% of their terrestrial lands as protected areas, and conservation and management costs for large protected areas would be high for many developing countries [20]. Thus, determining what areas contribute the most conservation value could be an alternative goal in these instances. In South Korea’s case, we found that expanding PAs to 15.3% of the mainland area (Fig 3a), for an overall PA extent of 14,587.55 km^2^, including existing PAs, would improve overall plant species’ range representation to a mean 16.8% for all species, to 30.2% for endangered species, to 22.4% for endemic species, and to 20.8% for biological resources species. By using the other scenario without locking in the existing PAs (Fig 3b), 13,197.35 km^2^ (13.9% of the mainland area) of PAs would improve overall plant range representation to a mean 13.5% for all species, and 15.3%, 14.4%, and 14.1% for endangered, endemic, and biological resources species, respectively.

Even though the additional PAs scenario that includes existing PAs represents higher percentages of species’ ranges, it is missing many biodiversity hotspots with high species richness in non-mountainous areas and in areas far from the existing PAs, such as the northern-central areas (S1 Fig). Lowland species have been identified as highly vulnerable to climate change [41], and therefore selected low elevation areas in the southern coastal areas are likely candidates for important conservation areas. In addition, southeastern areas could function as key areas for preserving coastal mountain species, which were also identified as climate vulnerable species in a previous study [41].

While an expansion of PAs from the commonly selected areas would not fully meet spatial goals of the Aichi convention, its species-protection intentions are likely more achievable, and by using the locations we identify could optimize conservation representation of plant species. However, since plant species are only one component of biodiversity, the PAs we identify may not be representative of overall biodiversity. Other taxonomic groups may need conservation in other areas, so additional studies using other groups could solve questions for national biodiversity conservation, and which might increase target conservation proportions to closer to the Aichi Biodiversity Targets. In addition, our analysis focused on optimizing the representation of plant species ranges, however some rare plants may occur in limited locations or stands which were not captured using a national-scale assessment approach and may need to be conserved in addition to the extents identified here.

Diverse physical environments support diverse species and may contribute to the persistence of the ecological and evolutionary processes that can maintain biodiversity [2,52]. Therefore, including diverse ecosystems in the network of PAs could efficiently help to protect biological diversity. Furthermore, coastal areas are especially valuable to protect because terrestrial, atmospheric, and marine systems interact, and transitions occur between adjacent ecological systems in the coastal areas [53].

### Limitations and future directions

Good-quality data on species distributions are required for species conservation planning. However, sufficient data are rarely available for a complete inventory of biodiversity [2]. This study used intensively surveyed plant data for 2,297 species, but many species had few records even though some are not rare, and about 44% of the known plant taxa do not appear at all. By deploying a multi-response SDM MARS, which takes advantage of occurrence data from other species, we were able to model 2,297 species’ potential range areas, including endangered species, at a grid resolution of 0.1 km^2^. This finer spatial resolution was useful for subsequent conservation assessments using Marxan on 4 km^2^ grid.

We recognize that SDM model quality is largely influenced by the number of records [43], and that species we modeled with few observation records generally produced range models with lower AUC scores. For example, there were 53 species that had AUC scores < 0.5, of which 30 species had two or three observation records (S1 Table). We opted to include these species for the following reasons. First, we wanted to have the largest number of species represented as possible, and while recognizing the risks to modeled range map accuracy, excluding them would prevent their presence in this conservation assessment. We compared the difference of the combined species richness maps of all species and of species had AUC scores ≥ 0.5. There was little difference in species richness patterns between the two maps, and the correlation coefficient between the two maps was 0.99 (S3 Fig). Second, there is a tradeoff in terms of temporal loss [54] in that the rapidly developing parts of South Korea could eliminate critical conservation areas from consideration if conservation planning is delayed until another national assessment is completed, and there is therefore an urgency to conduct as complete an assessment as possible, which is an additional motivation for inclusion of species with few records, rather than wait for additional surveys to increase the number of records. We conducted a limited sensitivity analysis by running the Marxan with species that had AUC scores ≥ 0.5, and found that the average percentage of species’ ranges captured in alternative PAs were very similar to the Marxan runs using all species (S6 Table).

The Third National Ecosystem Survey used for this analysis represents the best plant data currently available, but it still does not include records for 398 endemic, and 63 endangered species. This points to the need for further surveys, even while recognizing the tremendous effort put into the current data, to support systematic conservation planning at the national level.

On the other hand, as we tried to use as many species as possible in this study including rare species, data availability constrained us to focus mostly on climate variables for predicting species ranges, and we could not consider each species’ individual characteristics, such as biotic interactions, climate adaptability, and dispersal patterns in the modeling [55,56]. Also, we only considered plant species for identifying additional PAs. We did not constrain locations of potential PAs using other variables such as land use, land ownership, habitat condition, and opportunity costs for other land uses, which limit policy options in practice. Indeed, in the two alternative PAs scenarios, from 3.9–5.2% of selected lands have some level of urban land use already present. Site selection for additional PA implementation would need to include these considerations, as well as surveys to confirm actual presence, if specific endangered species are targeted.

In addition, many species are expected to shift their ranges in response to shifting climates and biogeochemical drivers [57]. Therefore, conservation planning also needs to consider the impacts of future climate scenarios [58,59]. Although this study only considered species’ current suitable environments, focusing on climate vulnerable species in designating additional PAs would be one of the climate mitigation activities for conserving ecosystems.

## Supporting information

S1 Table The list of modeled species. (CSV)

S2 Table Average percentage of species’ ranges captured in the existing protected areas and in equal-area alternative PAs scenarios. (PDF)

S3 Table Average percentage of species’ ranges captured in additional PAs including existing PAs scenarios. First sensitivity analysis to select species range representation target.
(PDF)

S4 Table Average percentage of species’ ranges captured in additional PAs including existing PAs scenarios. Second sensitivity analysis using the same species range conservation target to all species.
(PDF)

S5 Table Environmental variables used in the MARS SDM models. (PDF)

S6 Table Average percentage of species’ ranges captured in equal-area alternative PAs scenarios using species have AUC scores ≥ 0.5. We ran Marxan using range conservation targets for species with AUC scores ≥ 0.5.
(PDF)

S1 Fig Combined species richness maps from MARS SDM for each group category for South Korea. (a) All species, (b) Endangered species, (c) Endemic species, and (d) Biological resources species.
(PDF)

S2 Fig Average percentage of species’ ranges captured in additional PAs including existing PAs scenarios using the same species range conservation target to all species for: (a) all species, (b) endangered species, and (c) endemic species and biological resources species. (PDF)

S3 Fig Species richness of all species and of species have AUC scores > 0.5. (PDF)

S1 Appendix GIS raster layer for S1A Fig. The layer was projected using WGS 1984.
(TIF)

S2 Appendix GIS raster layer for S1B Fig. The layer was projected using WGS 1984.
(TIF)

S3 Appendix GIS raster layer for S1C Fig. The layer was projected using WGS 1984.
(TIF)

S4 Appendix GIS raster layer for S1D Fig. The layer was projected using WGS 1984.
(TIF)
