# Supplementary material for: Assessing shortfalls and complementary conservation areas for national plant biodiversity in South Korea
Source: PLoS One. 2018 Feb 23;13(2):e0190754. doi: 10.1371/journal.pone.0190754 (PMC5825007; doi:10.1371/journal.pone.0190754)
Supplement: S2 Table — (PDF) [file pone.0190754.s002.pdf]

S2 Table. Average percentage of species' ranges captured in the existing protected areas and in equal-area alternative PAs scenarios.

Scenario	Species range conservation target	Boundary length modifier	% of PAs to total land area	Mean % of all species' ranges inside	Mean % of endangered species' ranges inside	Mean % of endemic species' ranges inside	Mean % of biological resource species' ranges inside
Existing PAs	-	-	5.7	6.3	14.4	9.9	8.7
Equal-area alternative	10% of all species	0	5.1	5.9	5.3	5.1	5.2
		0.0007	5.0	4.3	4.4	4.1	4.2
Equal-area alternative	50% for endangered 30% for endemic and bio resource 10% of remaining	0	4.5	4.6	4.5	4.6	4.5
		0.0007	4.8	4.5	4.2	4.4	4.3
