# Supplementary material for: Assessing shortfalls and complementary conservation areas for national plant biodiversity in South Korea
Source: PLoS One. 2018 Feb 23;13(2):e0190754. doi: 10.1371/journal.pone.0190754 (PMC5825007; doi:10.1371/journal.pone.0190754)
Supplement: S3 Table — First sensitivity analysis to select species range representation target. (PDF) [file pone.0190754.s003.pdf]

S3 Table. Average percentage of species' ranges captured in additional PAs including existing PAs scenarios. First sensitivity analysis to select species range representation target.

Scenario	Species range conservation target	Boundary length modifier	% of PAs to total land area	Mean % of all species' ranges inside	Mean % of endangered species' ranges inside	Mean % of endemic species' ranges inside	Mean % of biological resource species' ranges inside
Same targets	10% of all species	0	16.7	17.8	27.4	21.8	20.5
		0.0007	16.6	18.2	31.8	23.9	22.3
	15% of all species	0	16.6	17.4	27.1	21.6	20.3
		0.0007	17.0	18.5	32.4	24.3	22.7
Specific targets for species groups	50% for endangered 30% for endemic and bio resource 10% of remaining	0	11.0	11.9	22.4	16.4	15.0
			16.5	17.3	27.3	21.6	20.3
		0.0007	15.2	16.6	29.9	22.1	20.6
			16.7	18.2	32.1	24.0	22.4
	55% for endangered 35% for endemic and bio resource 15% of remaining	0	11.0	11.8	22.3	16.4	15.0
			16.7	17.4	27.1	21.6	20.3
		0.0007	15.4	16.8	29.9	22.3	20.8
			16.8	18.4	31.9	24.0	22.5
