# Supplementary material for: Assessing shortfalls and complementary conservation areas for national plant biodiversity in South Korea
Source: PLoS One. 2018 Feb 23;13(2):e0190754. doi: 10.1371/journal.pone.0190754 (PMC5825007; doi:10.1371/journal.pone.0190754)
Supplement: S4 Table — Second sensitivity analysis using the same species range conservation target to all species. (PDF) [file pone.0190754.s004.pdf]

S4 Table. Average percentage of species' ranges captured in additional PAs including existing PAs scenarios. Second sensitivity analysis using the same species range conservation target to all species.

Scenario	Species range conservation target	Boundary length modifier	% of PAs to total land area	Mean % of all species' ranges inside	Mean % of endangered species' ranges inside	Mean % of endemic species' ranges inside	Mean % of biological resource species' ranges inside
Same targets	10% of all species	0.0007	15.4	16.9	30.0	22.4	20.8
			16.6	18.2	31.8	23.9	22.3
	15% of all species	0.0007	15.3	16.8	30.2	22.4	20.8
			17.0	18.5	32.4	24.3	22.7
	20% of all species	0.0007	14.3	15.6	28.7	21.1	19.5
			16.8	18.4	32.0	24.1	22.5
	25% of all species	0.0007	13.7	14.9	27.6	20.2	18.7
			16.9	18.4	32.1	24.1	22.5
	30% of all species	0.0007	13.3	14.4	26.9	19.6	18.2
			16.9	18.4	31.6	23.9	22.4
