# Supplementary material for: Assessing shortfalls and complementary conservation areas for national plant biodiversity in South Korea
Source: PLoS One. 2018 Feb 23;13(2):e0190754. doi: 10.1371/journal.pone.0190754 (PMC5825007; doi:10.1371/journal.pone.0190754)
Supplement: S5 Table — (PDF) [file pone.0190754.s005.pdf]

S5 Table. Environmental variables used in the MARS SDM models.

Environmental predictors	Minimum value	Maximum value	Model	Knot value
Annual mean temperature[1]	23	147	1	101.78
			2	97.48
			3	94.06
			4	85
			5	63.48
			6	85
			8	103.49
			9	92.97
			10	83.75
			11	90.25
			12	102
			14	103
			15	85
Elevation[2]	1	1895.271	1	612.22
			3	1321.97
			4	137.39
			8	327.03
			12	798.03
			14	296.97
Mean temperature of warmest quarter[1]	144	253	5	231
			7	217
			13	226.51
Annual precipitation[3]	993	1971	2	1143
			7	1687
Temperature seasonality[4]	7726	10504	7	9190
			13	9260
Mean temperature of coldest quarter[1]	-109	43	2	-20

[1] Scaling Factor: 10, Unit: Degrees Celsius; [2] Unit: Meters; [3] Unit: Milimeters;
[4] Scaling Factor: 100, Unit: Degrees Celsius
