# Supplementary material for: Assessing shortfalls and complementary conservation areas for national plant biodiversity in South Korea
Source: PLoS One. 2018 Feb 23;13(2):e0190754. doi: 10.1371/journal.pone.0190754 (PMC5825007; doi:10.1371/journal.pone.0190754)
Supplement: S6 Table — We ran Marxan using range conservation targets for species with AUC scores ≥ 0.5. (PDF) [file pone.0190754.s006.pdf]

S6 Table. Average percentage of species' ranges captured in equal-area alternative PAs scenarios using species have AUC scores ≥ 0.5. We ran Marxan using range conservation targets for species with AUC scores ≥ 0.5.

Scenario	Species range conservation target	Boundary length modifier	% of PAs to total land area	Mean % of all species' ranges inside	Mean % of endangered species' ranges inside	Mean % of endemic species' ranges inside	Mean % of biological resource species' ranges inside
Equal-area alternative	10% of all species	0	6.1	6.0	5.9	6.0	6.0
		0.0007	5.2	4.5	5.0	4.5	4.5
Equal-area alternative	50% for endangered 30% for endemic and bio resource 10% of remaining	0	5.7	5.7	5.9	5.8	5.8
		0.0007	5.9	5.3	8.0	5.9	5.7
