# Supplementary material for: Assessing shortfalls and complementary conservation areas for national plant biodiversity in South Korea
Source: PLoS One. 2018 Feb 23;13(2):e0190754. doi: 10.1371/journal.pone.0190754 (PMC5825007; doi:10.1371/journal.pone.0190754)

S1 Fig. Combined species richness maps from MARS SDM for each group category for South Korea: (a) All species, (b) Endangered species, (c) Endemic species, and (d) Biological resource species. The values in legend represent the number of species: greener areas have higher plant species richness in each group category.

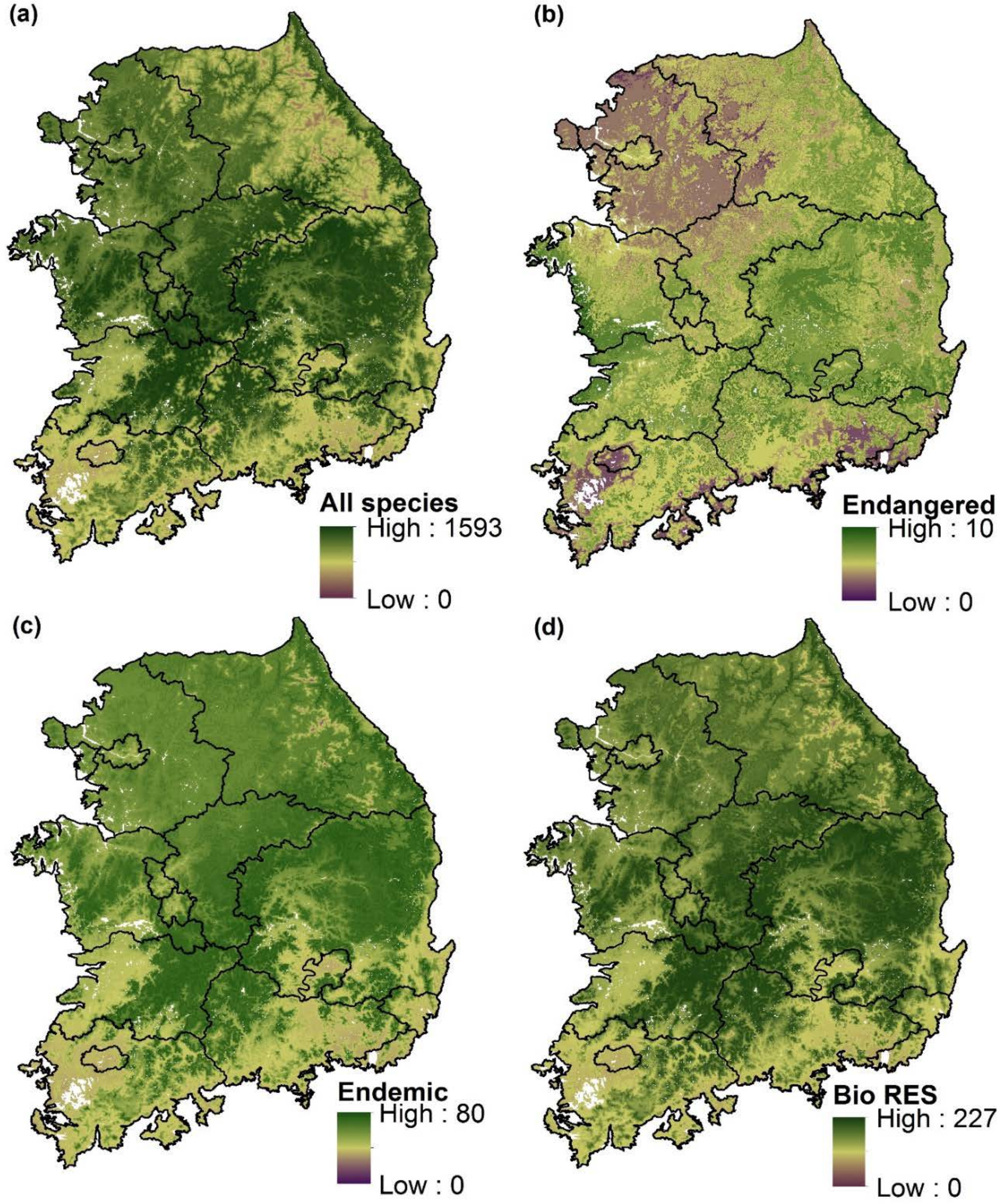

Supplement: S1 Fig — (a) All species, (b) Endangered species, (c) Endemic species, and (d) Biological resources species. (PDF) [file pone.0190754.s007.pdf]
