# Supplementary material for: Assessing shortfalls and complementary conservation areas for national plant biodiversity in South Korea
Source: PLoS One. 2018 Feb 23;13(2):e0190754. doi: 10.1371/journal.pone.0190754 (PMC5825007; doi:10.1371/journal.pone.0190754)

S2 Fig. Sensitivity analysis of species range representation targets. These charts relate the extent of PAs selected when different target percentages of species' range area were inputted into Marxan, and retaining the existing extent of PA's in the overall area selected. The average percentage of species' ranges captured is shown on the Y-axis, while the PA extent selected is shown as a percent of South Korea's land base on the X-axis. We selected the run that targeted 15% of all species ranges (and wound up capturing 16.8%) as the run that most closely approximated our scenario of doubling the extent of PAs, and being more implementable than the 17% of all land areas called for in the Aichi Biodiversity Convention. This model run is shown in red, with lines to axes to show the area and average percent of each species range captured. The four charts are based on the numbers in S4 Table, and represent the proportion of species' ranges captured for: (a) all species, (b) endangered species, (c) endemic species, and (d) biological resources species.

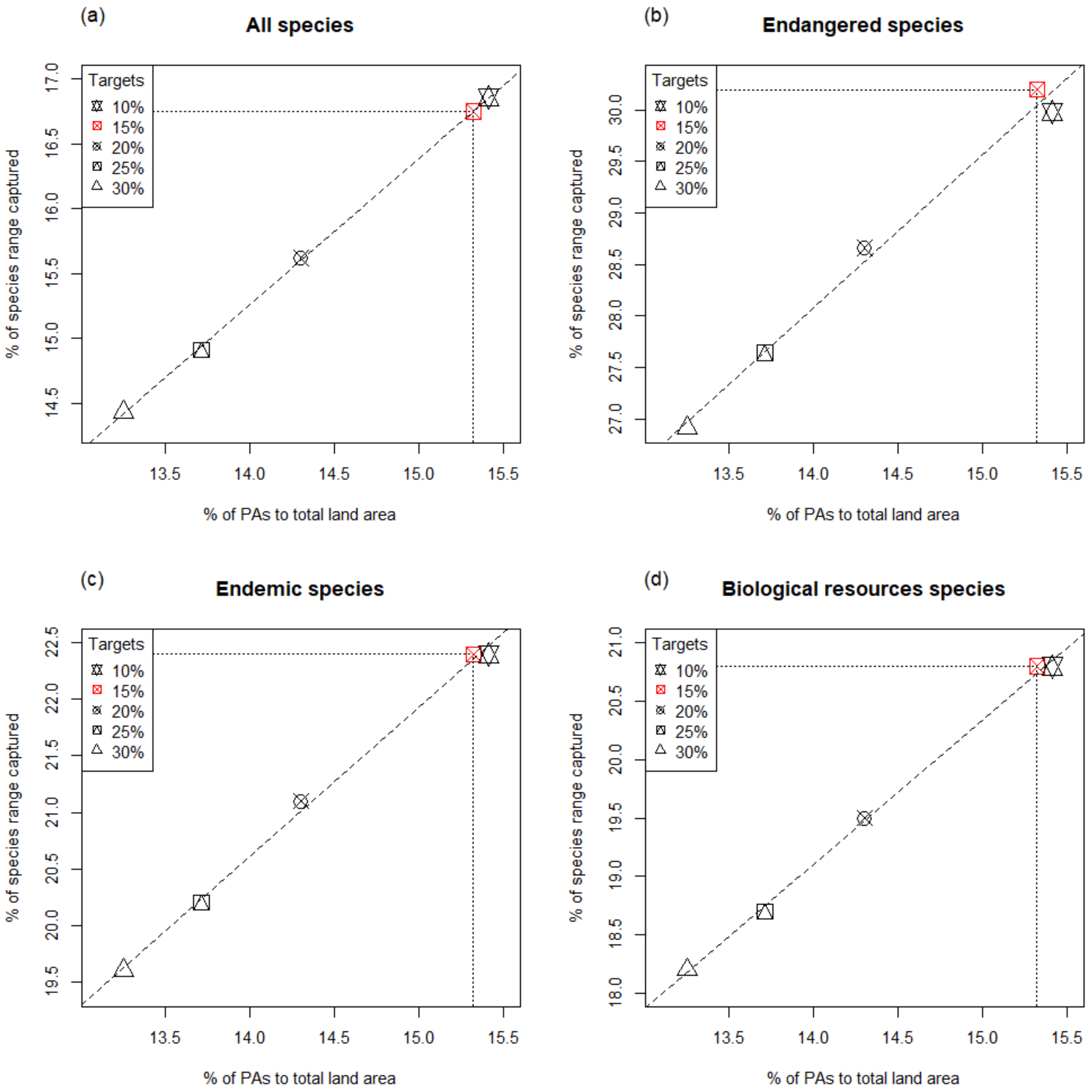

Supplement: S2 Fig — (PDF) [file pone.0190754.s008.pdf]
