# Supplementary figures and images for: Assessing shortfalls and complementary conservation areas for national plant biodiversity in South Korea
Source: PLoS One. 2018 Feb 23;13(2):e0190754. doi: 10.1371/journal.pone.0190754 (PMC5825007; doi:10.1371/journal.pone.0190754)

**S3 Fig. Species richness of all species (left) and of species have AUC scores > 0.5 (right).**

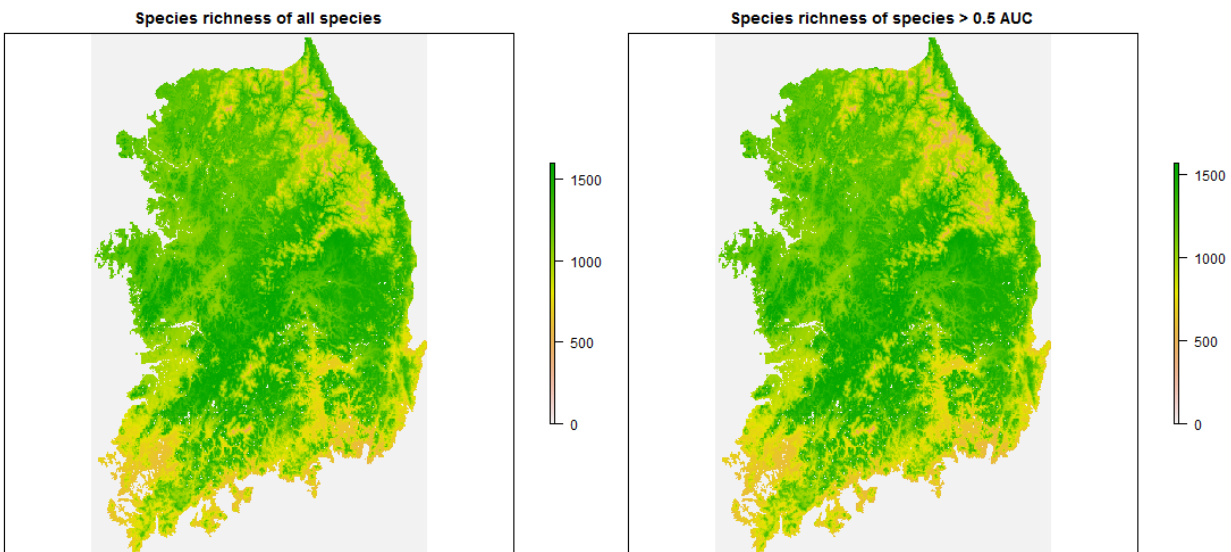

Supplement: S3 Fig — (PDF) [file pone.0190754.s009.pdf]
